# Preparation and Characterization of Strongly Sulfonated Acid Block and Random Copolymer Membranes for Acetic Acid Esterification with 2-Propanol

**DOI:** 10.3390/polym14132595

**Published:** 2022-06-27

**Authors:** Verónica Rosiles-González, Ronan Le Lagadec, Paulina Varguez-Catzim, María I. Loria-Bastarrachea, Abigail González-Díaz, Emanuel Hernández-Núñez, Manuel Aguilar-Vega, María Ortencia González-Díaz

**Affiliations:** 1 Laboratorio de Membranas, Unidad de Materiales, Centro de Investigación Científica de Yucatán, A.C., Calle 43 No. 130, Chuburná de Hidalgo, Mérida 97205, Mexico; very_1994@hotmail.com (V.R.-G.); paulivar41@gmail.com (P.V.-C.); marisa@cicy.mx (M.I.L.-B.); mjav@cicy.mx (M.A.-V.); 2 Instituto de Química, Universidad Nacional Autónoma de México, Circuito Exterior s/n, Ciudad Universitaria, Ciudad de México 04510, Mexico; ronan@unam.mx; 3 Instituto Nacional de Electricidad y Energías Limpias, Reforma 113, Col. Palmira, Cuernavaca 62490, Mexico; abigail.gonzalez@ineel.mx; 4 Centro de Investigación y de Estudios Avanzados del IPN, Unidad Mérida, Mérida 97310, Mexico; emanuel.hernandez@cinvestav.mx; 5 CONACYT—Centro de Investigación Científica de Yucatán, A.C., Calle 43 No. 130, Chuburná de Hidalgo, Mérida 97205, Mexico

**Keywords:** block copolymers, random copolymers, catalytic membranes, esterification, isopropyl acetate

## Abstract

In this paper, we report the synthesis of block and random copolymers of 2-acrylamido-2-methyl-1-propane sulfonic acid (AMPS) and methyl methacrylate (MMA), with different AMPS feed ratios. These solution-processable copolymers with strongly sulfonated acid groups resulted in membranes with tunable ion exchange (IEC) and water absorption capacities. AFM images confirmed the microphase separation of **PAMPS-*b*-PMMA-1:1** block copolymer membrane, annealed under the appropriate conditions. The resulting copolymers from the random combination of a 1:1 molar ratio of AMPS and MMA monomers are effective at enhancing the esterification conversion of acetic acid, when compared with a reaction catalyzed by PAMPS-*b*-PMMA block copolymers and the previously studied catalytic membranes. With the **PAMPS-*co*-PMMA-1:1** membrane, the esterification reaction using acetic acid achieved 85% isopropyl acetate. These results are closely correlated with the increase in IEC (2.63 mmol H^+^g^−1^) and the relationship between weight loss (20.3%) and swelling degree (68%) in 2-propanol.

## 1. Introduction

Over the years, polymeric membrane technology has been successfully applied in fuel cells, natural gas purification, gas water treatment, hydrogen recovery, and drug delivery systems that involve separation or transport processes [1,2,3]. Recently, catalytic membranes have been used in processes that involve chemical reactions wherein separation of the reaction products may or may not take place, such as in dehydrogenation reactions, selective oxidation, methane-steam reforming, the Fischer-Tropsch reaction, dimerization, and esterification and transesterification reactions [4,5,6,7]. In the field of esterification and transesterification reactions, catalytic membranes present the advantage of combining chemical conversion and molecular separation in one step, thereby reducing the separation cost [8] and presenting a low environmental impact [9]. Several solid acids and ionic polymers, such as zirconium sulfate (Zr(SO_4_)_2_), sulfonated polyethersulfone (SPES)/phosphotungstic acid (PWA), poly(styrene sulfonic acid) (PSSA), poly(2-acrylamido-2-methyl-1-propane sulfonic acid) (PAMPS), sulfonated polyphenyl sulfone (SPPS) or sulfonated polyether sulfone (SPES) with different sulfonation degrees (SD), embedded or blended in a polymeric matrix of poly(vinyl alcohol) (PVA), polyacrylonitrile (PAN), polyether sulfone (PES) or polyphenyl sulfone (PPS), have been explored as catalytic membranes for esterification and transesterification reactions [10,11,12,13,14,15,16,17,18]. Most of these catalytic membranes are blends of at least two different polymers that present a decrease in their catalytic stability on reusing the materials, which is attributed to the loss of solid acid or ionic polymer from the polymeric matrix [14]. Thus, the development of functional membranes with strong acid groups that allow higher catalytic efficiency without catalytic activity loss due to reuse is now required, to prepare new heterogeneous acid catalysts. Within this context, copolymers (either block or random) with rationally designed chemical structures could be considered new alternatives that are able to overcome the shortcomings of blends to form a catalytic membrane via these reactions. Both random and block copolymers are expected to prevent the loss of ionic polymers, which occurs with blending in the polymeric matrix due to the strong covalent bonds that link together the monomers in the macromolecule, increasing the membrane’s useful life and reusability in esterification/transesterification reactions. Moreover, in the case of block copolymers, it seems plausible that they are able to form micro-channels within the polymeric structure that will promote more effective transport of the reagents through the membrane.

Isopropyl acetate (IPOAc) is commercially produced for use in a wide range of chemical applications. It is used as an industrial solvent for synthetic resins and polymers such as vinyl copolymers, acrylics, polyesters, nitrocellulose, ethyl cellulose, epoxy resins, methacrylic resins, polyamides, and alkyds, and as a solvent in adhesive production, as well as in printing inks and as an aroma compound in perfumes [19,20]. Isopropyl acetate is produced in the liquid-phase esterification reaction of acetic acid with 2-propanol, in the presence of homogeneous or heterogeneous acidic catalysts [20]. In an industrial process, the unreacted acetic acid is recirculated in order to increase the overall yield, and the separation of the reaction products is carried out by reactive distillation, which involves high energy consumption [21]. The use of catalytic membranes as heterogeneous catalysts in the acetic acid (HOAc) esterification reaction emerges as an attractive alternative that reduces the production cost due to energy savings, the elimination of the catalyst separation step, and because it is an environmentally friendly process. For example, Zhang et al. [22] used a catalytically active membrane with a composite structure of PVA/PES and ion-exchange resins in the esterification of acetic acid and *n*-butanol, reaching 71.9% and 91.4% conversion in a batch and pervaporation reactors at 85 °C, respectively. Caetano et al. [23] reached ~90% biodiesel conversion in the esterification of palmitic acid with methanol, catalyzed by a PVA membrane crosslinked with sulfosuccinic acid (PVA_SSA40) using a 1:63 alcohol:acid molar ratio at 60 °C. Recently, Wang et al. [24] elaborated a novel, highly porous membrane by crosslinking PVA with 5-sulfosalicylic acid. This membrane was used as a catalyst in the esterification of acetic acid and ethanol in a pervaporation membrane reactor (PVMR), and an acetic acid conversion of 98.4% was reached at 75 °C in 12 h. A new type of catalytic composite membrane, containing a support layer of commercial porous PAN, a middle layer of a dense sodium alginate (SA)/MoS_2_, and an upper layer of a PVA/p-hydroxybenzene sulfonic acid (Pha) blend was reported by Liu et al. [25]. The authors reported that the acetic acid conversion rate reached 94.3% under assisted pervaporation at 75 °C in 12 h, which is 29% higher than that of the batch reaction.

Thus, in the present work, we synthesized a series of block and random copolymers of 2-acrylamido-2-methyl-1-propane sulfonic acid (AMPS) and methyl methacrylate (MMA) with different AMPS feed ratios. In these copolymers, strongly acidic poly(2-acrylamido-2-methyl-1-propanesulfonic acid) (PAMPS) is expected to catalyze the esterification reaction, whereas poly(methyl methacrylate) (PMMA), due to its hydrophobic nature should control water swelling of PAMPS [20]. Physicochemical characterization and tests of the ion exchange capacity (IEC) and absorption capacity of PAMPS*-b*-PMMA and PAMPS-*co*-PMMA as copolymers membranes were carried out, and their performance as heterogeneous catalysts on the acetic acid esterification with 2-propanol in a batch reactor are discussed.

## 2. Materials and Methods

### 2.1. Materials and Reagents

First, 2-acrylamido-2-methylpropane sulfonic acid (AMPS, 99%), sodium 2-acrylamido-2-methylpropanesulfonate (AMPSA, 50 wt% in H_2_O), methyl methacrylate (MMA, 99%), water (HPLC), potassium persulfate (KPS, ≥99.0%), methanol (MeOH, 99.9%), N,N-dimethylformamide (DMF, 99.8%), ethyl 2-bromoisobutyrate (EBiB, 98%), 2-propanol (99.8%) and 1,10-phenanthroline (phen) were purchased from Aldrich, Toluca (Edo. México) México. Acetic acid (HOAc, 99.9%) was supplied by Fermont, Monterrey (Nuevo León) México. MMA was purified by passing it through an inhibitor remover column. The [Ru(*o*-C_6_H_4_-2-py)(phen)(MeCN)_2_]PF_6_ catalyst was synthesized as previously reported elsewhere [26].

### 2.2. Synthesis of Block and Random Copolymers 

For both copolymers (block and random), the [AMPS]_o_: [MMA]_o_ feed molar ratios were 1:1 and 1:2.

#### 2.2.1. Synthesis of PAMPS-*b*-PMMA 

**Scheme 1:**
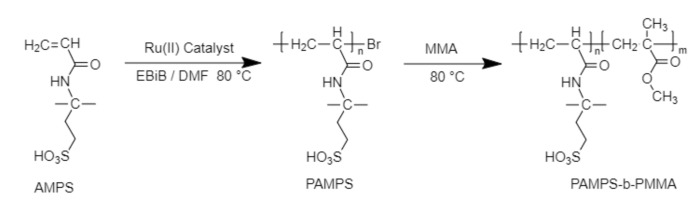
Synthesis of PAMPS-*b*-PMMA by ATRP, catalyzed by a Ru(II) complex.

The block copolymers were synthesized by radical polymerization, catalyzed by a ruthenium complex (Scheme 1), as reported previously by Martínez-Cornejo et al. [27]. In a typical synthesis, a 25-mL Schlenk flask was charged with AMPS (0.39 g, 1.88 mmol), [Ru(*o*-C_6_H_4_-2-py)(phen)(MeCN)_2_]PF_6_ (12.5 mg, 0.0185 mmol) and EBiB (2.75 μL, 0.0185 mmol), in 0.5 mL DMF at 80 °C for 16 h. A known amount of previously degassed MMA (1.88 or 3.76 mmol) was then added to the reaction mixture using a syringe under a N_2_ purge and the solution was heated again at 80 °C for 20 h. The resultant copolymer was poured into diethyl ether and the product obtained thereby was filtered. After dissolving in dichloromethane, the polymeric solution was purified through a silica gel column.

#### 2.2.2. Synthesis of PAMPS-*co*-PMMA

**Scheme 2:**
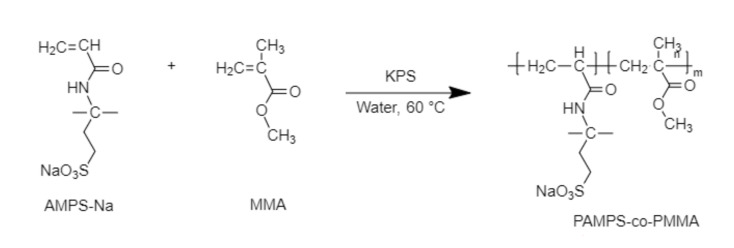
Synthesis of PAMPS-*co*-PMMA by free radical polymerization.

The random copolymers were synthesized by free radical polymerization, using potassium persulfate (KPS) as an initiator. As reported by Shen et al. [28], AMPS-Na (3.68 mL, 9.68 mmol), KPS (0.3 mol %), a known amount of MMA (9.68 or 19.3 mmol), and previously deoxygenated water (0.5 mL) were introduced to a 50-mL Schlenk tube. The homogeneous mixture was degassed and the reaction tube was immersed in an oil bath at 60 °C. Polymerization was stopped after 2 h, and the reaction mixture was precipitated into ethanol. Finally, the copolymers were dried at 80 °C under vacuum for 24 h (see Scheme 2).

### 2.3. Preparation of Dense Membranes

The dense membranes were prepared by the casting method, using 5% solutions of the copolymers. Two different solvents were used: methanol to dissolve PAMPS-*b*-PMMA and N,*N*-dimethylformamide (DMF) for PAMPS-*co*-PMMA. The solutions were poured into an aluminum ring, then the solvent was slowly evaporated at 30 and 80 °C, respectively. The membranes were dried under vacuum at 80 °C for 24 h. Finally, the PAMPS-*co*-PMMA membranes were immersed in a 1 M HCl methanol solution for 24 h to convert the sulfonate groups to acid.

### 2.4. Characterization

The ^1^H-NMR analyses were performed on a Varian NMR-600 spectrometer (Agilent technologies, Santa Clara, CA, USA), using CD_3_OD for PAMPS-*b*-PMMA and DMF-d_7_ for PAMPS-*co*-PMMA. An Agilent 1100 series GPC-SEC (Agilent technologies, Santa Clara, CA, USA), equipped with two columns (Zorbax 60-S and 1000-S) and a RI detector, was used to measure the molecular weights. A mobile phase (flow rate of 0.7 mL min^−1^) of DMF with 0.05 % lithium bromide (LiBr) and PMMA standards was used for the analysis. A TA Instrument DSC 2920 (Columbus, OH, USA) calorimeter was used to evaluate the glass transition temperatures of the polymers at a heating rate of 10 °C/min, in a nitrogen atmosphere, between 50 °C and 200 °C. The surface morphology of the block copolymer membrane was analyzed with a tapping-mode atomic force microscope (AFM) using a Bruker Multimode 8 instrument (Bruker, Billerica, MA, USA).

The ion exchange capacity (IEC, mmol H^+^g^−1^) was measured using a titration method. First, 100 mg of membrane samples that had previously been dried were immersed in NaOH solution (0.1 M, 5 mL) at room temperature for 24 h [28]. Thereafter, the remaining solution was titrated with 0.02 N HCl, using phenolphthalein as an indicator. The IEC value was calculated by:

(1) $$\mathrm{IEC}\;\left({\mathrm{mmolg}}^{-1}\right)=\frac{M_{\mathrm{NaOH}}V_{\mathrm{NaOH}}-M_{\mathrm{HCl}}V_{\mathrm{HCl}}}{W_{s}}\;$$

where V_NaOH_ and M_NaOH_ are the NaOH volume consumed and the respective molarity; V_HCl_ and M_HCl_ are the volume and molarity of the HCl consumed in the titration, respectively; and W_s_ is the membrane sample weight. 

Solvent uptake and weight loss: Dry membrane samples were immersed in 2-propanol or methanol (5 mL) for 5 days at 60 °C. The samples were blotted with filter paper and then weighed every 24 h. Finally, after 5 days, the samples were dried at 60 °C under vacuum for 24 h and weighed. The 2-propanol or methanol uptake (%) and % weight loss (WL) were calculated by:

(2) $$\mathrm{Solvent}\;\mathrm{uptake}\;\left(\%\right)=\frac{m-m_{o}}{m_{o}}\;\times \;100\;$$

(3) $$\mathrm{WL}=\frac{m_{o}-m_{s}}{m_{o}}\times 100$$

where m − m_0_ is the weight difference between the swollen mass and initial mass of the membrane sample, and m_s_ is the dry sample mass after 2-propanol uptake.

### 2.5. Esterification Reaction Performance of Copolymer Membranes

The esterification reaction of acetic acid was carried out in a series of 12 mL screw-cap vials under stirring at 60 °C. In each vial, 20 mg of membrane, cut into small pieces, was immersed in 3 mL of 2-propanol for 24 h. The esterification reaction was then initiated with the addition of 0.25 mL of acetic acid; it was continued up to a reaction time of 29 h. Vials were removed at specified intervals and the titration technique with 1 M NaOH was used to quantify the esterification reaction kinetics. The conversion (C) to isopropyl acetate (IPOAc) was calculated using Equation (4) [22,29]:

(4) $$C=\left(1-\frac{n_{t}}{n_{o}}\right)\times 100$$

where *n_o_* is the initial number of CH_3_COOH moles and *n_t_* is the number of CH_3_COOH moles at time *t*.

## 3. Results and Discussion

### 3.1. Synthesis and Characterization of PAMPS-b-PMMA

**Figure 1:**
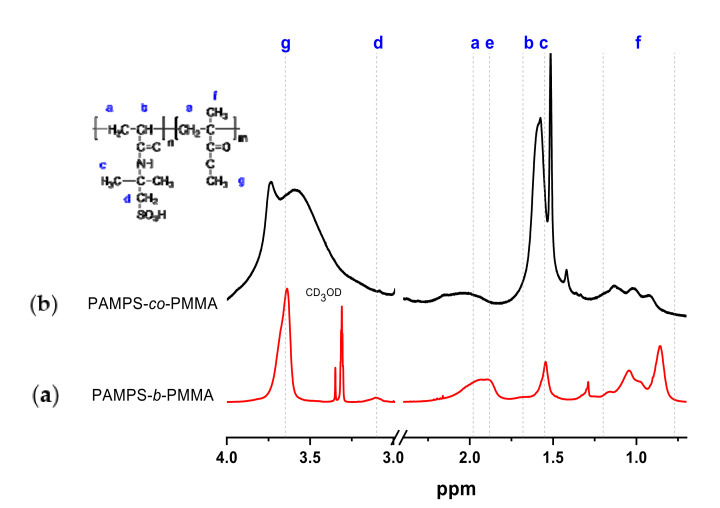
(**a**) ^1^H-NMR spectrum of PAMPS-*b*-PMMA in CD_3_OD and (**b**) PAMPS-*co*-PMMA in DMF-d_7_. Molar composition [AMPS]_o_/[MMA]_o_ = 1/2.

The PAMPS-*b*-PMMA copolymers were synthesized by atom transfer radical polymerization (ATRP), catalyzed by a ruthenium (II) complex. The chemical structure and composition of the block copolymers were confirmed by ^1^H-NMR, where the spectra (Figure 1a) exhibit the characteristic signals of each block (PMMA and PAMPS). Four signals at 1.54, 1.68, 1.93, and 3.10 ppm correspond to polymer backbone protons (a, b), methyl protons (c), and to methylene protons (d) adjacent to the acid group, respectively [27,30]. Moreover, with the incorporation of PMMA as a block, the copolymer shows the presence of broad peaks between 1.10 and 0.80 ppm (f) and around 3.62 ppm (g) due to main-chain protons [31]. The PAMPS-*b*-PMMA molar composition was calculated by the ^1^H-NMR integration ratio of the peaks, at 3.10 ppm from –CH_2_- (d) of PAMPS and at 3.62 ppm from –CH_3_ (g) of PMMA. In this case, the molar compositions calculated by ^1^H-NMR were lower than the theoretical ones. For block copolymers with monomer concentrations and feed [AMPS]_o_: [MMA]_o_ molar ratios of 1:1 (50/50 mol %) and 1:2 (33/67 mol %), the copolymer molar compositions found by NMR were 24/76 and 12/88 mol %, respectively, which is attributed to the fact that a low concentration of PAMPS macroinitiator chains allows the growth of a second PMMA block, due to the incompatibility of a purely hydrophilic ionic block (PAMPS) with the purely hydrophobic nature of PMMA [32].

**Figure 2:**
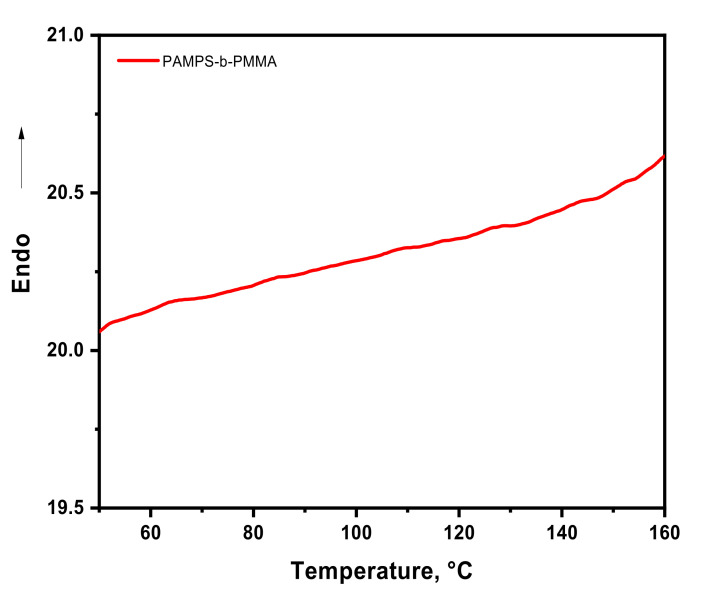
The DSC second-heating curve of PAMPS-*b*-PMMA copolymer.

Two different glass transition temperatures (T_g_), associated with individual polymer PAMPS (124 °C) and PMMA (114 °C) blocks, were expected to be observed in the DSC curves, due to their inherent immiscibility [33,34]. However, as can be seen in Figure 2, no clear T_g_ was presented by the PAMPS-*b*-PMMA copolymers, which may be due to the large number of water molecules bound to the copolymer chains [34,35], the presence of chain interactions or crosslinking between the different functional groups, [28] and/or the close values of their T_g_s. 

**Figure 3:**
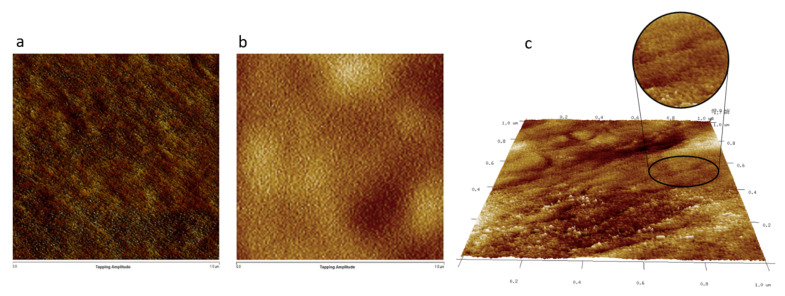
AFM images of the **PAMPS-*b*-PMMA-1:1** membrane: (**a**) thermally annealed at 120 °C for 72 h, (**b**) microwave-annealed for 180 s in the presence of toluene solvent, and (**c**) the 3-dimensional texture of the image in (**b**).

The immiscibility of the amphiphilic block copolymers leads to microphase separation, where well-defined nanostructures can be obtained under the appropriate conditions [36,37]. In this context, the resultant morphologies of the block copolymer membrane (with 24% PAMPS and roughly 90 µm membrane thickness) were investigated after a prolonged or fast annealing step on silicon surfaces. Figure 3 shows the tapping-mode AFM images of **PAMPS-*b*-PMMA-1:1** (24/76 mol%) when thermally annealed under vacuum at 120 °C for 72 h (Figure 3a) and when annealed using a microwave reactor for 180 s at 120 °C in a toluene environment (Figure 3b,c), as reported by B. Xiaojiang et al. [37]. 

The membrane surface (Figure 3a) exhibits a microphase separation structure, where the paler regions are due to the hydrophobic component, while the darker regions represent the hydrophilic PAMPS domains, which appear dark because they contain a high water fraction [38]. However, a well-defined morphology was not obtained in the **PAMPS-*b*-PMMA-1:1** membrane annealed at 120 °C. Only segregated domains were observed, which was to be expected since it is difficult to obtain a completely ordered state by thermal annealing [39]. Meanwhile, the microwave-annealed **PAMPS-*b*-PMMA-1:1** membrane (Figure 3b) clearly shows an array of almost hexagonally perforated layer (HPL) morphology. This ordered morphology is obtained due to the blocks’ composition (f_PAMPS_ = 0.25), as well as the tendency of sulfonated block copolymers to produce hexagonally packed cylinders when exposed to a toluene environment [40,41]. These results confirm that PAMPS-*b*-PMMA block copolymers were obtained.

### 3.2. Synthesis and Characterization of PAMPS-co-PMMA

On the other hand, it is well known that polymers must have the appropriate properties for the fabrication of polymeric membranes for a particular application. For example, in the case of catalytic membranes for esterification or transesterification reactions, it is desirable for the polymers to present a high concentration of active sites (acid or base), adequate swelling properties, and membrane-forming ability, as well as high molecular weight, among other properties [18,20,27,42,43]. Thus, for comparison, random copolymers with the same theoretical molar composition were synthesized using the free radical polymerization technique; Figure 1 provides a representative ^1^H-NMR spectrum for each block and random copolymer with the highest PMMA concentration. The free-radical polymerization of AMPS with MMA using potassium persulfate (KPS) as an initiator in an aqueous medium was not successful. Therefore, it was necessary to use AMPS as its deprotonated sodium salt (AMPSA) and, subsequently, to protonate the formed copolymer to obtain PAMPS-*co*-PMMA. The ^1^H-NMR signals corresponding to each proton in the PAMPSA-*co*-PMMA were observed in the spectra (Figure 1b), which became broader and shifted slightly downfield when compared with PAMPS-*b*-PMMA. The molar composition could not be estimated by the integration ratio because of the overlapping between the aforementioned signals at 3.10 ppm (d) of PAMPS and at 3.62 ppm of (g) of PMMA. However, according to the IEC values, the theoretical composition in the feed agrees with that of the experimental one, as will be discussed later. In comparison with PAMPS-*b*-PMMA, PAMPS-*co*-PMMA random copolymers with higher PAMPS concentrations turn out to be insoluble in methanol. For this reason, DMF-d_7_ or DMF was used for their characterization and membrane preparation, respectively. 

An important difference between random and block copolymers was found in their molecular weights and polydispersity index (*Ð*) values (see Table 1). The number-average molecular weights (M_n_) and *Ð* values for block copolymers PAMPS-*b*-PMMA were in the range of 68,000–78,500 and 1.45–1.48, respectively, whereas for PAMPS-*co*-PMMA random copolymers, these values increased by a factor of 5.4 and 5.8, with *Ð* values ranging from 1.87 to 1.91. The reason for this behavior is that high molecular-weight polymers are formed immediately in free-radical polymerization and remain unchanged during the polymerization process, whereas in a living polymerization, such as ATRP, the number-average molecular weight increases linearly with conversion, leading to narrow molecular weight distribution [44].

However, while both copolymers are capable of membrane-forming behavior, more flexible and practical membranes were obtained with high-molecular-weight PAMPS-*co*-PMMA copolymers.

### 3.3. Ion Exchange Capacity (IEC) and Swelling Degree

Ion exchange capacity (IEC), which indicates the total number of active sites in the membrane that may be able to catalyze a reaction, and swelling degree, which allows reactants to access sulfonic acid groups, are important parameters for evaluating the catalytic performance of a membrane [18,20,42]. The IEC value depends on the amount of PAMPS in the copolymer. The block copolymers with 12 and 25% PAMPS exhibited IEC values of 0.55 and 1.40 mmol H^+^g^−1^, respectively, whereas in the random copolymers, the IEC values were 2.63 and 1.57 mmol H^+^g^−1^ (see Table 1). These latter values corresponded to a PAMPS composition of 50 and 34% in the copolymers, which agrees with the theoretical feed molar ratios of [AMPS]_o_: [MMA]_o_ of 1:1 and 1:2 respectively. The IEC values of **PAMPS-*b*-PMMA-1:1**, **PAMPS-*co*-PMMA-1:1** and **PAMPS-*co*-PMMA-1:2** membranes were similar to those reported for a transesterification catalytic reaction for methyl-ester formation, ranging between 1.26 and 3.80 mmol H^+^g^−1^, and were higher than those for a Nafion-117 membrane (0.92 mmol H^+^g^−1^) [7,12,18,20,42].

**Figure 4:**
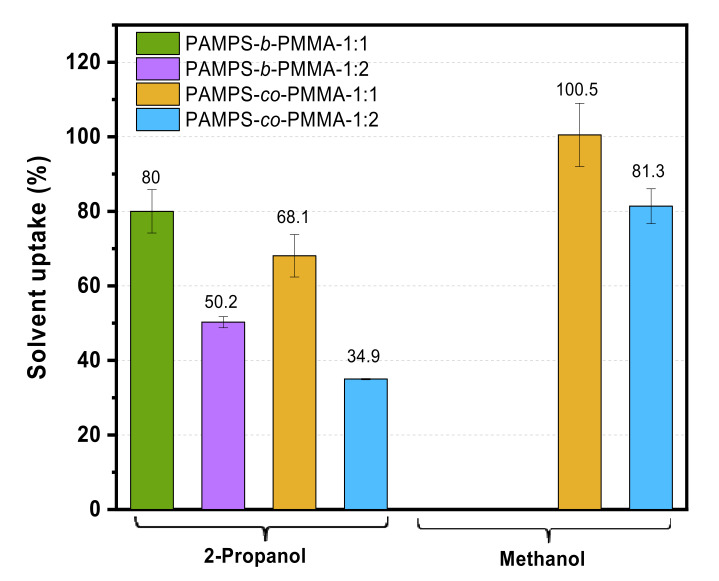
The 2-propanol and methanol uptake of membranes made from PAMPS*-b*-PMMA and PAMPS-*co*-PMMA copolymers at different PAMPS concentrations.

**Figure 5:**
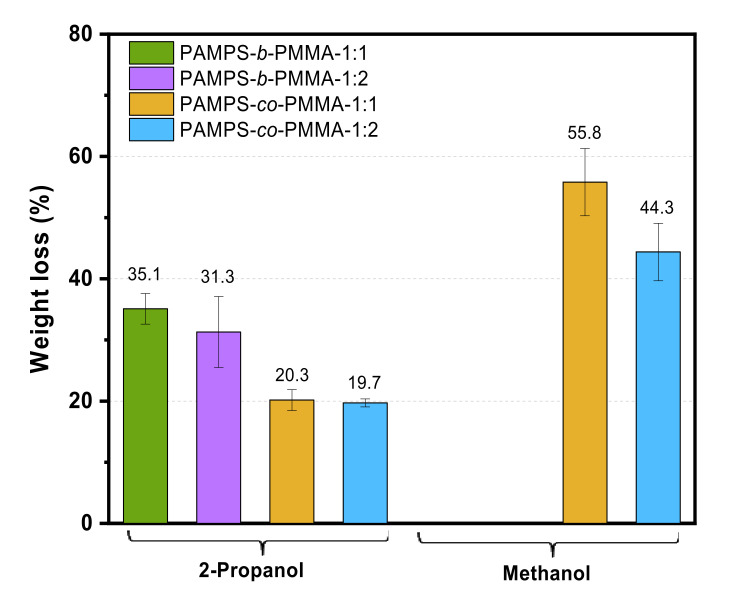
Weight loss percentages for membranes PAMPS-*b*-PMMA and PAMPS-*co*-PMMA in 2-propanol and methanol.

Figure 4 and Figure 5 show the swelling degree and weight loss values for PAMPS-*b*-PMMA and PAMPS-*co*-PMMA membranes. In addition, 2-propanol uptake values of 80% and 50.2% were found for membranes **PAMPS-*b*-PMMA-1:1** and **PAMPS-*b*-PMMA-1:2**, respectively, due to the high hydrophilicity of PAMPS. These results indicate that 2-propanol uptake increases with the increase of PAMPS concentration in the copolymer. Both the PAMPS-*b*-PMMA membranes were completely soluble in methanol. It can also be seen that the stability of the membranes cast from PAMPS-*co*-PMMA increased when they were immersed in 2-propanol or methanol (see Figure 3), which is due to the high content of random sulfonic groups in the polymeric chain, which allows a certain degree of physical crosslinking via hydrogen bonding between the sulfonic functional groups present. In fact, the swelling degree of **PAMPS-*co*-PMMA-1:1** and **PAMPS-*co*-PMMA-1:2** diminishes considerably in 2-propanol, in comparison to membranes prepared with block copolymers. Moreover, the PAMPS-*co*-PMMA membranes were insoluble in methanol, presenting 100.5% and 81.3% methanol uptake, respectively. In comparison, the crosslinked PVA/PAMPS catalytic membranes reported by Corzo-González et al., prepared using poly(vinyl alcohol) (PVA) and PAMPS blends that are similar to PAMPS-*co*-PMMA random copolymer membranes, presented a methanol uptake increase of 50–60% with increasing PAMPS concentration from 10 to 30% in the blend [20]. This result confirms that the presence of the random sulfonic group in the material allows a certain degree of physical crosslinking that increases membrane stability in solution [45].

Moreover, as can be seen in Figure 5, the weight losses (WL, %), after 2-propanol immersion for 5 days, were 20.17 ± 1.7 and 19.7 ± 0.6 for the **PAMPS-*co*-PMMA-1:1** and **PAMPS-*co*-PMMA-1:2** membranes, respectively, whereas membranes made from PAMPS-*b*-PMMA copolymers presented WL values of 35.1 ± 2.5 and 31.3 ± 5.8, with feed [AMPS]_o_: [MMA]_o_ molar ratios of 1:1 and 1:2, respectively. The high weight-loss values in block copolymer membranes are attributed to their lower molecular weight, in comparison with those obtained in random copolymers. PAMPS-*b*-PMMA turned out to create fragile and slightly brittle membranes when submerged in 2-propanol, making it difficult to extract samples from the solvent for weighing in the WL experiment. Finally, the PAMPS-*co*-PMMA membranes presented WL values of around 50% after methanol immersion.

Due to their high IEC and degree of swelling in 2-propanol, as well as an optimal relationship between weight loss in 2-propanol and IEC, the **PAMPS-*co*-PMMA-1:1** and **PAMPS-*b*-PMMA-1:1** membranes were chosen to study their catalytic activity in the esterification reaction of acetic acid with 2-propanol. 

### 3.4. Catalytic Performance in the Esterification Reaction

**Figure 6:**
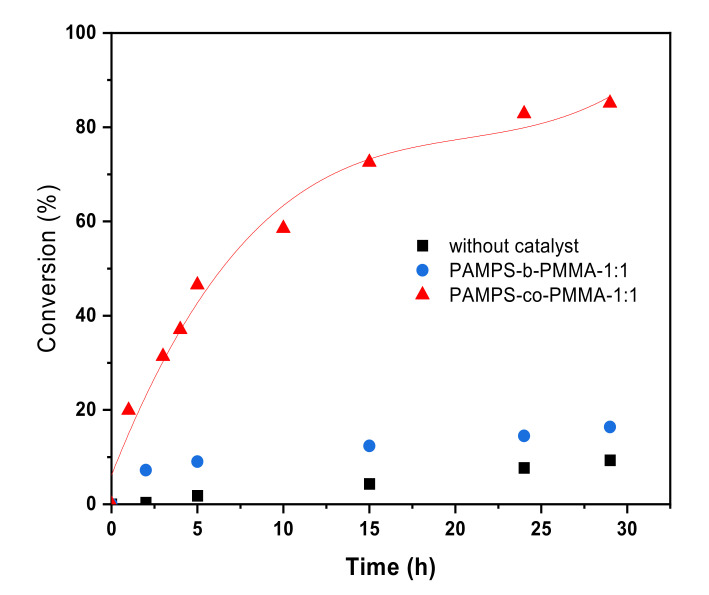
Isopropyl acetate conversion versus reaction time for PAMPS-*b*-PMMA-1:1 (▲), PAMPS-*co*-PMMA-1:1 (●), and without catalyst (■), at 60 °C for 29 h.

Figure 6 shows the kinetic reaction of the acetic acid esterification catalyzed by **PAMPS-*co*-PMMA-1:1** or **PAMPS-*b*-PMMA-1:1**. In addition, the reaction was performed without a catalyst. It was observed that the esterification reaction proceeds even in the absence of the catalyst, due to the weak acidity of acetic acid. However, it barely reaches a 9% conversion in 29 h. Conversely, 17% isopropyl acetate conversion was obtained with the **PAMPS-*b*-PMMA-1:1** membrane in the same period of time, despite its high IEC value (1.40 mmol H^+^g^−1^) and swelling degree (80%) in 2-propanol that allow the diffusion of the reactants to access sulfonic groups for catalyzing the esterification reaction. The low isopropyl acetate conversion of the **PAMPS-*b*-PMMA-1:1** membrane could be attributed to its poor stability in solution and its high weight loss in 2-propanol (35.1%), making it difficult for it to absorb the moisture produced by the esterification reaction [46]. 

On the other hand, in the presence of a **PAMPS-*co*-PMMA-1:1** catalytic membrane, the conversion is greatly accelerated compared to the **PAMPS-*b*-PMMA-1:1** membrane, reaching 85% in 29 h. The result can be ascribed to the high IEC value (2.63 mmol H^+^g^−1^) and adequate 2-propanol uptake (68%), which allow reactants to diffuse through the swollen membrane and access the acid groups. Figure 6 shows a rapid isopropyl acetate conversion in the first 15 h, up to 75%. After that, there is a stabilization period in the following 14 h, with isopropyl acetate conversion at between 75 and 85%. The efficiency of **PAMPS-*b*-PMMA-1:1** as a catalytic membrane in acetic acid esterification is expected to be enhanced in a hybrid process involving a catalytic reaction and pervaporation for reaction product extraction [22]. 

Table 2 shows the **PAMPS-*co*-PMMA-1:1** catalytic performance for esterification, in comparison with Amberlyst and various PVA-based catalytic membranes [22,23,46,47]. Amberlyst 15 presented a slightly lower conversion rate (78%) at 75 °C in 29 h [46] than was observed for **PAMPS-*co*-PMMA-1:1** at 60 °C. The membrane used by Neguyen et al. [47] was based on poly(styrene sulfonic acid) in a PVA matrix, whereas the second membrane used PVA as a polymeric matrix crosslinked with sulfosuccinic acid (SSA) [23]. Both PVA membranes also presented a slightly lower conversion rate (70% and 83% respectively) than **PAMPS-*co*-PMMA-1:1**. If we compare these with a PVA/PES membrane embedded with ion-exchange resin, this clearly shows a lower conversion rate, even though the reaction was carried out at 75 °C. 

Overall, the **PAMPS-*co*-PMMA-1:1** catalytic membrane evinces a more efficient performance than a PVA/PSSH catalytic blend membrane [47], a PVA/PES membrane embedded with ion-exchange resin [22], and PVA cross-linked with sulfosuccinic acid, (SSA) [23] in terms of esterification reactions.

## 4. Conclusions

A series of block and random copolymers of MMA with different AMPS feed ratios were successfully synthesized via atom transfer radical polymerization (ATRP) catalyzed by a ruthenium(II) complex and free radical polymerization, respectively. Although these copolymers presented good film-forming ability, the PAMPS-*co*-PMMA random copolymers were more flexible and versatile membranes, due to their high molecular weight in comparison with block copolymers. PAMPS-*co*-PMMA membranes presented the highest IEC values (1.57–2.63 mmol H^+^g^−1^), with 2-propanol uptake of between 34 and 68%, whereas PAMPS-*b*-PMMA exhibited IEC values and 2-propanol uptake in the range of 0.55–1.40 mmol H^+^g^−1^ and 50–80%, respectively. These results are well correlated with the increase in the molar composition of PAMPS in the copolymer. It was observed that the catalytic activity performance of the **PAMPS-*co*-PMMA-1:1** membrane, as a heterogeneous catalyst in acetic acid esterification with 2-propanol at 60 °C, is more effective than in the membranes obtained with block copolymers. A conversion rate of 85% was obtained with this **PAMPS-*co*-PMMA-1:1** membrane, which is expected to be enhanced in a hybrid process involving reaction and separation in one single unit.

## Figures and Tables

**Table 1:** Composition and molecular weights of block and random copolymers.

Copolymer	PAMPS (%mmol) ^a^	PMMA (%mmol) ^a^	M_n_ × 10^−4^ (gmol^−1^) ^b^	M_w_ × 10^−4^ (gmol^−1^) ^b^	Ð ^b^	IEC ^c^ (mmol H^+^g^−1^)
**PAMPS-** ***b*** **-PMMA-1:1**	24	76	7.85	11.6	1.48	1.40
**PAMPS-** ***b*** **-PMMA-1:1**	12	88	6.80	9.86	1.45	0.55
**PAMPS-** ***co*** **-PMMA-1:1**	-	-	42.5	81.2	1.91	2.63
**PAMPS-** ***co*** **-PMMA-1:1**	-	-	39.7	74.2	1.87	1.57

^a^ Molar composition, estimated by ^1^H-NMR; ^b^ IEC values, expressed as mmol H^+^g^−1^ of membrane; ^c^ molecular weights.

**Table 2:** Esterification performance by Amberlyst and PVA-based acid catalytic membranes, in comparison with the **PAMPS-*co*-PMMA-1:1** membrane reported in this work.

Material	Ref.	T (°C)	IEC mmol H^+^g^−1^	Raw Material	Conversion (%)	Time(h)
Amberlyst 15	46	75	4.75	Acetic acid/2-propanol	78	29
PVA/PSSH	47	50	-	Propanoic acid/propanol	70	25
PVA_SSA40	23	60	-	Oleic acid/methanol	~83	25
PVA/PES/Ion-Exchange resin	22	75	-	Acetic acid/n-butanol	68.2	20
**PAMPS-** ***co-*** **PMMA-1:1**	This work	60	2.63	Acetic acid/2-propanol	85.1	29

PVA: polyvinyl alcohol; SSA: sulfosuccinic acid; PSSH: poly(styrene sulfonic acid); PES: polyethersulfone.
